# Genomic Signatures of Somatic Mutation and Selection Shape Distinct Clonal Lineages in *Bougainvillea × buttiana* ‘Miss Manila’ Bud Sport

**DOI:** 10.3390/genes17040471

**Published:** 2026-04-17

**Authors:** Hongyan Meng, Qun Zhou, Duchao Chen, Bayan Huang, Mingqiong Zheng, Wanqi Zhang

**Affiliations:** 1 Fujian Institute of Subtropical Botany, Xiamen 361006, China; mhy1984@126.com (H.M.);; 2 National Bougainvillea Germplasm Resource Bank, Xiamen 361006, China; 3 Xiamen Botanical Garden, Xiamen 361006, China; 4 Xiamen Chaozhiran Horticulture Co., Ltd., Xiamen 361000, China; 5 Xiamen Weiguanshu Biotech Co., Ltd., Xiamen 361006, China

**Keywords:** *bougainvillea*, bud sport, genotyping-by-sequencing (GBS), population genomics, genetic differentiation, selection signal, somatic mutation

## Abstract

**Background/Objectives**: Bud sports (somatic mutations) offer a quick way to develop new *bougainvillea* varieties by altering specific traits while keeping the desirable genetic background of the original cultivar. However, we still lack a comprehensive understanding of their genomic architecture and the molecular mechanisms behind their formation. This study aimed to characterize the population genomic characteristics of bud sports derived from the commercial variety *Bougainvillea × buttiana* ‘Miss Manila’. **Methods**: We employed genotyping by sequencing (GBS) on 39 accessions, including 27 bud sports and 12 conventional varieties. Population genomic analyses, such as principal component analysis (PCA), phylogenetic reconstruction, ADMIXTURE, and diversity statistics (π, He, Tajima’s D), were performed on 64,810 high-quality SNPs. Genome-wide scans for differentiation (FST) and selective sweeps (XP-CLR) were also conducted. **Results**: Bud sports showed significantly lower genetic diversity (π and He) than conventional varieties, which matches their clonal origin. PCA, phylogenetic, and ADMIXTURE analyses (optimal K = 4) revealed clear genetic differentiation and distinct population structures between the two groups. The bud sport population possessed fewer private alleles and a less negative Tajima’s D value. Genomic scans identified regions under selection in bud sports, with functional annotation pointed to genes involved in ubiquitin-mediated proteolysis and RNA transport. Notably, *Bou_119143* (UDP-rhamnose rhamnosyltransferase 1) showed a high mutation frequency specifically in bud sports. **Conclusions**: We provide the first population-genomic evidence that bud sports of ‘Miss Manila’ are genetically distinct clonal lineages, shaped by somatic mutation and selection. These findings support bud sports as efficient sources for germplasm innovation. The identified genomic regions and candidate genes lay a foundation for future marker-assisted selection and molecular breeding in *bougainvillea*.

## 1. Introduction

The global ornamental horticulture industry thrives on continuous innovation and the introduction of new plant varieties, with genetic diversity serving as the cornerstone for breeding cultivars that have enhanced aesthetic value, environmental resilience, and market appeal [1]. *Bougainvillea*, a quintessential tropical and subtropical genus, has cemented its status as a landscape and pot plant staple worldwide, widely celebrated for its vividly colored bracts, prolonged flowering period, and remarkable drought tolerance. The continuous enhancement of its commercial value largely depends on the ongoing selection of new varieties with novel bract colors, variegated foliage, or compact plant architecture, which in turn drives the further optimization and innovation of breeding techniques [2].

In *bougainvillea* breeding practices, sexual hybridization and somatic bud sports are two major sources of variation [2]. Traditional breeding predominantly relies on sexual hybridization, a technique that integrates favorable genetic material from different parents via meiotic recombination. Indeed, the three fundamental species of *bougainvillea*, namely *Bougainvillea glabra*, *Bougainvillea spectabilis*, and *Bougainvillea peruviana*, along with their hybrid derivatives *Bougainvillea × buttiana*, *Bougainvillea × spectoperuviana*, and *Bougainvillea × spectoglabra*, form the genetic framework of the hundreds of horticultural varieties available today [3,4]. Hybridization can aggregate superior genes from different parents in terms of bract color, plant architecture, and stress resistance, thus achieving unprecedented trait combinations [2,5]. However, hybrid breeding also has significant limitations, such as a lengthy juvenile phase, highly heterozygous genetic backgrounds, difficulties in genetically dissecting complex traits, and the tendency to break up well-adapted gene combinations in segregating progeny [4,6].

In contrast, selection based on somatic mutations (bud sports) offers an efficient alternative route. Bud sports can directly generate specific trait variations while fully preserving the desirable comprehensive traits and genetic background of the original cultivar, enabling one-step variety improvement [5,7,8]. This effectively overcomes the issues of trait segregation and long breeding cycles typical of sexual reproduction. For example, in *bougainvillea*, important cultivars like ‘Mrs. Butt’ have given rise to several new varieties, including ‘Scarlet Queen’, through a series of bud sports, greatly enriching bougainvillea’s varietal diversity [9,10].

In recent years, thanks to the rapid development of genomic technologies, significant breakthroughs have been made in *bougainvillea* research. The completion of a chromosome-level reference genome for *B. glabra* has provided a crucial foundation for analyzing its biological characteristics at the molecular level [11]. Furthermore, large-scale genome sequencing of the hybrid cultivar *B. × buttiana* ‘Mrs Butt’ revealed whole-genome duplication events during its evolution, providing valuable clues for understanding cultivar origin and the betalain pigment metabolism pathway [12]. Moreover, population genomics studies, using reduced-representation genome sequencing on 84 *bougainvillea* accessions, not only classified them into six genetic subgroups (B1–B6) but also detected extensive gene flow between these subgroups. Through selective sweep analysis, these studies further found that metabolic pathways related to defense response and cell division regulation (such as terpenoid and triterpenoid biosynthesis, glycerophospholipid metabolism, etc.) were under significant selection in bud sport varieties, offering new perspectives for revealing the molecular mechanisms of bud sport emergence and the domestication trajectory of *bougainvillea* [13].

Despite significant progress in existing research, most current work still focuses on macro-level genetic diversity analysis among species or varieties. There remains a lack of systematic and in-depth research on the internal genomic structure of bud sport populations derived from a single variety, the fine-scale genetic differentiation between these bud sports and conventional cultivars, the selection signals driving the formation of bud sport traits, and the underlying key genes.

To address the aforementioned questions, this study established a population of 39 Bougainvillea accessions for systematic analysis. The population comprised two parts: a core group of 27 bud sport mutants derived from the commercial variety *B. × buttiana* ‘Miss Manila’ and a control group of 12 representative cultivars obtained through conventional breeding. Genotyping-by-sequencing (GBS) technology was employed to generate 64,810 high-quality SNP markers, and a systematic analysis was conducted on this basis on the genomic architecture, selection signals, and genetic differentiation characteristics of the bud sport population. This study reveals, for the first time from a population genomics perspective, the genetic nature of *bougainvillea* bud sports, providing not only a theoretical basis for bud sport breeding but also laying the foundation for precision breeding via marker-assisted selection and key gene cloning.

## 2. Materials and Methods

### 2.1. Plant Materials

A total of 39 *Bougainvillea* accessions were analyzed in this study (Table S1). The collection was comprised exclusively of two forms of this variety. The experiment consisted of 27 somatic mutants (bud sports) derived from the commercial cultivar *B. × buttiana* ‘Miss Manila’, labeled S1 to S27. These mutants exhibited stable phenotypic divergence from the wild-type cultivar, mainly in floral color intensity, bract hue, and leaf morphology, and were collected from diverse cultivation areas across the Philippines, Malaysia, and China (Taiwan, Fujian, Guangdong, and Yunnan provinces/regions). For each mutant accession, leaf samples were collected from three individual plants (clonal replicates) grown at the same site, and these replicates were pooled prior to DNA extraction to minimize within-accession variation. The control group comprised 12 conventional breeding cultivars, including representative accessions of *B. spectabilis*, *B. glabra*, and *B. × buttiana* hybrids. All control accessions are phenotypically stable, non-mutant genotypes with no documented bud sport variation, and were selected to represent the broader genetic background of cultivated bougainvillea for population comparison. The 12 control accessions were chosen to cover the major phylogenetic lineages of cultivated bougainvillea.

All plant materials were obtained from the National Germplasm Repository for Bougainvillea, Xiamen Botanical Garden (Xiamen, Fujian, China), where they are preserved as long-term living collections under standardized horticultural management. No wild populations were included in this study. Consequently, no physical voucher specimens have been deposited in a public herbarium. Given that all accessions are maintained as living clones with detailed records of origin, propagation history, and taxonomic identification, no formal herbarium vouchers were deposited. Taxonomic identification was performed and validated by Prof. Kefu Huang, a senior taxonomic expert affiliated with the National Germplasm Repository for Bougainvillea, following the classification standards documented in the “Flora of China” [14].

For DNA extraction, fully expanded young leaves were sampled from each accession in December 2023. For each accession, three individual plants (where available) were sampled, and their leaf tissues were pooled in equal mass before freezing. Leaf tissues were immediately frozen in liquid nitrogen and stored at −80 °C until genomic DNA isolation, minimizing degradation and ensuring consistent sampling quality across all accessions.

### 2.2. DNA Extraction and Genotyping-by-Sequencing (GBS)

Genomic DNA was isolated from young leaf tissue using the DNAsecure Plant Kit (TIANGENBiotech Co., Ltd., Beijing, China) according to the manufacturer’s instructions. DNA quality and concentration were evaluated via 1% agarose gel electrophoresis, NanoDrop spectrophotometry (Thermo Fisher Scientific, Wilmington, DE, USA), and Qubit 4 Fluorometry (Thermo Fisher Scientific, Wilmington, DE, USA). Only high-quality DNA samples (concentration > 50 ng/µL, OD260/280 ≈ 1.8–2.0, OD260/230 ≈ 2.0–2.5) were used for further analysis.

Library preparation was performed using the Super-GBS protocol [15]. Briefly, 100 ng of genomic DNA from each sample was digested with *PstI*-HF and *MspI* restriction endonucleases (New England Biolabs, Ipswich, MA, USA) at 37 °C for 2 h, followed by enzyme inactivation at 65 °C for 20 min. Following digestion, sample-specific barcoded adapters (Illumina) were ligated to the fragments using T4 DNA ligase (New England Biolabs, USA). Fragments of 300–700 bp were size-selected using AMPure XP magnetic beads (Beckman Coulter, Brea, CA, USA) and then amplified by PCR for 18 cycles. The final qualified libraries were pooled and sequenced on an Illumina NovaSeq 6000 platform (Illumina, San Diego, CA, USA) to generate 150 bp paired-end reads.

### 2.3. SNP Calling and Filtering

Raw sequencing reads were demultiplexed according to barcode sequences, and low-quality reads (Q-value < 20), adapter sequences, and short reads (<50 bp) were filtered using Fastp v0.20.0 [16]. Given the availability of the reference genome for *B. × buttiana* ‘Mrs. Butt’, a reference-guided SNP discovery pipeline was implemented in Stacks v2.4 [17]. The gstacks and populations modules were run with default parameters except for **--min-mapq 20** and **--min-clipping-coverage 5**. Only biallelic SNPs were retained. This process involved building a catalog of consensus loci from all samples and then genotyping each sample against this catalog. The initial raw SNPs were stringently filtered using Vcftools v0.1.16 [18] with the following criteria: loci had to be diploid, possess a total read depth (DP) ≥ 4, a Minor Allele Frequency (MAF) ≥ 0.01, and a genotyping missing rate < 20%. All other Vcftools filters were left at default settings. The final filtered SNP dataset was used for all subsequent population genetic analyses.

### 2.4. Population Genetic Analysis

Genetic relationships among the 39 accessions were investigated using multiple complementary approaches. A neighbor-joining (NJ) phylogenetic tree was constructed based on genetic distances with 1000 bootstrap replicates using Treebest v1.9.2 [19] and visualized with FigTree v1.4.4 [20]. Principal component analysis (PCA) was performed on the SNP matrix using GCTA v1.26.0 [21] with default parameters (no LD pruning) to visualize genetic clustering, and the results were visualized in R v4.3.1. Population structure was inferred using ADMIXTURE v1.3.0 [22] with the number of ancestral populations (K) set from 1 to 10. ADMIXTURE was run using the default block relaxation algorithm; for each K, ten independent replicates with different random seeds were performed, and the replicate with the highest likelihood was retained. Convergence was assumed when the log-likelihood change between iterations was <0.0001. The optimal K value was determined by identifying the lowest cross-validation (CV) error across ten replicates.

Genetic diversity parameters, including expected heterozygosity (He), observed heterozygosity (Ho), polymorphism information content (PIC), and nucleotide diversity (Pi), were calculated using the R package genepop v4.8.5 [23]. Genetic differentiation between populations was quantified by the pairwise fixation index (Fst) using the R package StAMPP v1.6.3 [24]. The number of private alleles between the two populations was calculated using the pegas v1.4 package in R [25]. Statistical differences in these parameters between populations were assessed via a *t*-test with a significance level of α = 0.05.

### 2.5. Selection Signal and Neutrality Test Analysis

Selective sweep regions between the two populations were detected using XP-CLR v1.0 software [26], with the conventional population as the reference population and the bud mutation population as the target population. The parameter settings were as follows: window size = 50 kb, step size = 10 kb, and maximum linkage disequilibrium (LD) distance = 50 kb. The XP-CLR score for each window was calculated, and windows with the top 5% of scores were defined as potential selective sweep regions.

Tajima’s D neutrality test was used to analyze the evolutionary dynamics of the populations. Tajima’s D values were calculated separately for the two populations (window size: 50 kb, step size: 10 kb) using the pegas v1.4 package in R [25] with default parameters for handling missing data; windows with more than 50% missing sites were excluded. 1000 simulation tests were performed to evaluate the significance of deviations from neutrality. Differences in Tajima’s D values between the two populations were compared via an independent-samples two-tailed Welch’s *t*-test with a significance level of α = 0.0001.

### 2.6. Functional Enrichment and Differential Mutation Gene Analysis

Using “mutation frequency of bud mutation population−mutation frequency of conventional population” as the indicator, differential analysis was performed with the R package DESeq2 v1.40.2 [27]. Although DESeq2 was originally developed for RNA-seq count data, it was applied here to biallelic SNP mutation frequencies (presence/absence of the mutant allele) after verifying that the frequency values approximated a negative binomial distribution (dispersion = 0.28). The screening criteria were set as follows: false discovery rate (FDR) < 0.01 and absolute value of mutation frequency difference > 0.5. FDR correction was performed using the Benjamini–Hochberg procedure.

KEGG pathway enrichment analysis was conducted on the differentially mutated genes using the R package clusterProfiler v4.8.3 [28], with the KEGG annotation information of the *Bougainvillea* reference genome as the background. The enrichment significance threshold was set at FDR < 0.05, and the Benjamini–Hochberg method was again applied for multiple testing correction across pathways. Bar plots of the top 10 significantly enriched pathways were generated.

Gene Set Enrichment Analysis (GSEA) was performed using the gseKEGG function of the R package clusterProfiler v4.8.3. First, “difference in mutation frequency between the bud mutation population and the conventional population” was used as the gene ranking indicator to construct a gene ranking list. With the KEGG pathway gene sets of the *Bougainvillea* reference genome as the background (consistent with the KEGG enrichment analysis), the number of permutations was set to 1000 and the significance threshold to FDR < 0.05.

## 3. Results

### 3.1. Sequencing Data Statistics and SNP Discovery

Reduced-representation genome sequencing (RRGS) was performed on 39 germplasm samples of *B. × buttiana* ‘Miss Manila’, including 27 bud sport varieties and 12 conventional non-sport varieties, using the Illumina NovaSeq PE150 platform. A total of 275,325,940 raw reads were obtained, corresponding to 40.48 Gb of raw base pairs (ranging from 0.63 to 2.63 Gb per sample). Following stringent quality control, which included the removal of adapter sequences, low-quality reads, and reads with excessive ambiguous bases, 261,217,220 clean reads (33.96 Gb of clean bases) were retained. The average proportion of clean reads was 83.55% (range: 80.83–87.04%), yielding an average of 0.67 Gb of high-quality data per sample, sufficient for subsequent genetic analyses (Table S2). Quality assessment indicated high data reliability, with an average GC content of 41.55% (range: 39.05–48.21%), Q20 of 96.96% (range: 95.27–97.84%), and Q30 of 91.98% (range: 88.22–93.96%). Subsequent SNP calling and filtering identified 64,810 high-confidence SNPs.

### 3.2. Genetic Diversity of SNP Markers and Population Comparison

**Figure 1:**
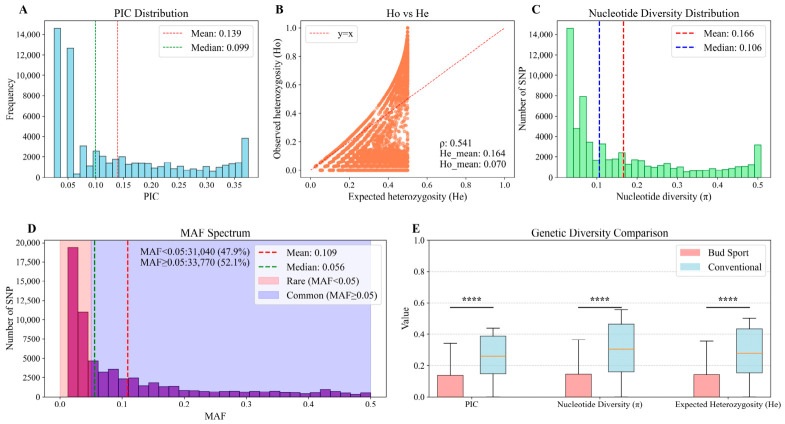
Genetic diversity analysis of 39 *B. × buttiana* ‘Miss Manila’ variants. (**A**) Distribution of the Polymorphic Information Content (PIC) values across the analyzed genome-wide markers. The red and green vertical dashed lines represent the mean and median PIC values, respectively. (**B**) Comparison of observed heterozygosity (Ho) and expected heterozygosity (He) among the variants. Orange points represent individual SNPs, and the red dashed line indicates the *y = x* reference line. (**C**) Genome-wide distribution of nucleotide diversity (π). (**D**) Minor Allele Frequency (MAF) spectrum of all identified SNPs. The light pink background indicates rare variants (MAF < 0.05), and the light blue background indicates common variants (MAF ≥ 0.05). The red and green vertical dashed lines represent the mean and median MAF values, respectively. (**E**) Comparative summary of key genetic diversity parameters, including nucleotide diversity (π), expected heterozygosity (He), and Polymorphic Information Content (PIC), between the bud sport (pink boxes) and conventional (light blue boxes) populations. Statistical significance (****) indicates *p* < 0.0001.

Genetic diversity was assessed across all 39 germplasm accessions using the 64,810 high-quality SNPs. The Polymorphism Information Content (PIC) and nucleotide diversity (π) both exhibited right-skewed distributions, with mean values of 0.139 and 0.166, respectively, indicating a preponderance of loci with low to moderate polymorphism (Figure 1A,C). The right-skewed distribution suggests that most SNP loci are under weak selection or neutral evolution, whereas a small fraction of highly polymorphic loci may be under balancing selection or located in genomic regions with higher mutation rates [29]. The comparison between expected heterozygosity (He, mean = 0.164) and observed heterozygosity (Ho, mean = 0.070) revealed that Ho was consistently lower than He across most loci (Figure 1B), a pattern that typically indicates population substructure or inbreeding, consistent with the clonal propagation and limited recombination in cultivated bougainvillea [30]. The Minor Allele Frequency (MAF) spectrum showed a balanced composition, with 47.9% of SNPs classified as low-frequency variants (MAF < 0.05) and 52.1% as common variants (MAF ≥ 0.05) (Figure 1D). The substantial proportion of low-frequency variants suggests the presence of rare alleles that may have arisen from recent somatic mutations or have been maintained by genetic drift in the cultivated germplasm.

Comparative analysis between the bud sport population (*n* = 27) and the conventional population (*n* = 12) revealed significantly reduced genetic diversity in the former. The bud sport group showed markedly lower values (*p* < 0.001) for nucleotide diversity (π), expected heterozygosity (He), and PIC compared to the conventional group (Figure 1E). This consistent reduction across multiple metrics indicates a narrower genetic base in the bud sports, consistent with their origin from somatic mutations of a single progenitor cultivar.

### 3.3. Population Genetic Structure

**Figure 2:**
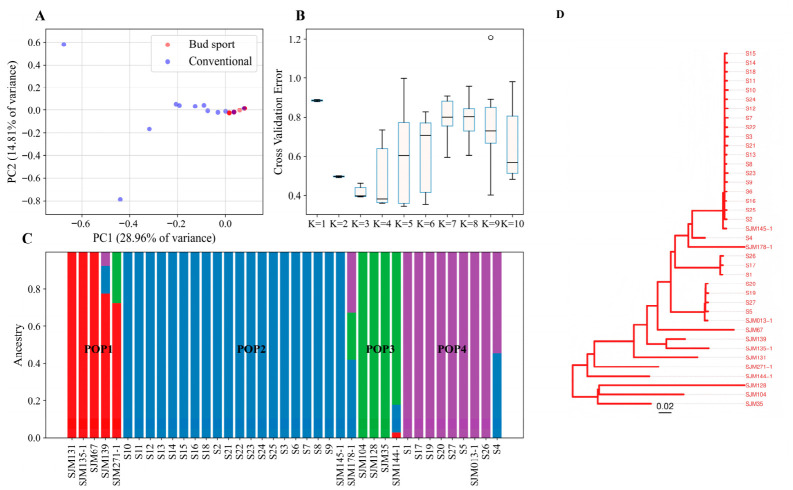
Population genetic structure of *B. × buttiana* ‘Miss Manila’ variants. (**A**) Principal components analysis (PCA) plot based on genome-wide SNPs. The first two principal components (PC1 and PC2) are shown, illustrating the genetic relationship and stratification among individuals. Red circles represent bud sport accessions, and blue circles represent conventional varieties. (**B**) Determination of the optimal genetic cluster number (K). Cross-validation (CV) error across a range of K values (from 1 to 10) from the ADMIXTURE analysis. The K value with the minimum CV error (K = 4) was selected as optimal. (**C**) Population structure bar plot inferred by ADMIXTURE at the optimal K = 4. Each vertical bar represents an individual, and the colored segments represent the estimated proportion of ancestry from each of the four genetic clusters (POP1–POP4, indicated by different colors). (**D**) Phylogenetic tree constructed from genome-wide SNPs, depicting the evolutionary relationships among the *B. × buttiana* ‘Miss Manila’ accessions.

The population genetic structure of the *B. × buttiana* ‘Miss Manila’ varieties was investigated using multiple complementary approaches. Principal component analysis (PCA) revealed clear genetic separation between the sport mutants and conventional varieties (Figure 2A). The first two principal components explained 28.96% (PC1) and 14.81% (PC2) of the total genetic variation, respectively. whereas the conventional varieties showed a dispersed distribution, the sport mutants formed a tight, distinct cluster, indicating substantial genetic differentiation between the two groups.

To determine the optimal number of genetic clusters, we performed a population structure analysis using ADMIXTURE with K values ranging from 1 to 10. The cross-validation error reached a minimum at K = 4 (Figure 2B), indicating that four ancestral populations best explained the genetic structure. At this optimal K value, the analysis identified four distinct genetic clusters (POP1-POP4; Figure 2C). Conventional varieties were primarily assigned to POP1 and POP3, while sport mutants predominantly constituted POP2 and POP4. Notably, two conventional accessions (SJM145-1 and SJM013-1) showed substantial ancestral components from POP2 and POP4, respectively, suggesting potential phylogenetic affinities with the sport groups.

The genetic partitioning observed in the structure analysis was further supported by a neighbor-joining phylogenetic tree (Figure 2D), which exhibited a topology highly consistent with the ADMIXTURE results. The sport mutants and conventional varieties formed distinct clades, with the sport mutants primarily clustering into two branches corresponding to POP2 and POP4 from the structure analysis. This bifurcation within the bud sport population suggests that at least two independent somatic mutation lineages have been maintained under cultivation, each giving rise to distinct sets of horticultural traits.

Collectively, these analyses demonstrate a well-defined genetic structure and significant divergence between the sport mutant and conventional variety groups, supporting their distinct genetic origins and evolutionary histories.

### 3.4. Characterization of Bud Sport-Specific Mutations

**Figure 3:**
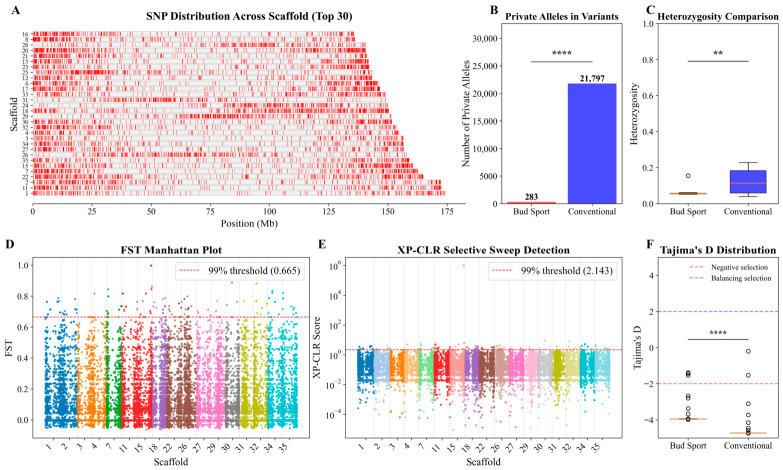
Genomic landscape of bud sport mutations and signatures of selection. (**A**) Genome-wide distribution of single-nucleotide polymorphisms (SNPs) across all scaffolds, illustrating the density and genomic context of genetic variations. Red bars represent the positions of SNPs along each scaffold. (**B**) Comparison of private alleles between the bud sport and conventional populations. The bar chart shows a significantly lower proportion of private alleles in the bud sport group (****, *p* < 0.0001). (**C**) Comparison of heterozygosity levels between the bud sport and conventional groups. Bud sports exhibit a significant change in heterozygosity (**, *p* < 0.01), demonstrating the impact of mutations on genetic diversity. (**D**) Manhattan plot of population differentiation (FST) between bud sport and conventional populations. Each colored dot represents an SNP, with different colors indicating distinct scaffolds. The red dashed horizontal line indicates the genome-wide significance threshold. (**E**) Results of the Cross-Population Composite Likelihood Ratio (XP-CLR) test, identifying genomic regions that have undergone selective sweeps in the bud sport lineage. Each colored dot represents a genomic window, with different colors indicating distinct scaffolds. The red dashed horizontal line indicates the genome-wide significance threshold. (**F**) Comparison of Tajima’s D values in bud sport (red) and conventional (blue) populations. The comparison reveals a significantly different allele frequency spectrum between the two groups (****, *p* < 0.0001), reflecting distinct demographic or selective histories.

The distribution of high-quality SNPs was analyzed across the top 30 longest scaffolds, providing broad genomic coverage despite an uneven distribution with enrichment in specific regions (Figure 3A). The uneven SNP density may reflect variation in recombination rates or selection pressures across the genome, with higher density in genic regions potentially associated with adaptive traits [31].

Analysis of private alleles revealed a substantial disparity between the populations (Figure 3B). The conventional population contained 23,797 private alleles, vastly outnumbering the 335 found in the bud sport population (*p* < 0.0001). This difference reflects their distinct histories: the bud sports, derived clonally from a single progenitor, possess a limited and recent gene pool, restricting the accumulation of private alleles. In contrast, the conventional varieties represent a more diverse genetic background. The near absence of private alleles in the bud sport population indicates that recent somatic mutations have contributed few novel alleles that are unique to this group, consistent with the fact that most phenotypic variation in sports arises from epigenetic changes or point mutations in a small number of genes rather than large-scale allele turnover.

Consistent with this, the average heterozygosity of the bud sport population was significantly lower than that of the conventional population (*p* < 0.01; Figure 3C), a direct consequence of the founder effect and clonal propagation, which limit the introduction of new genetic variation.

### 3.5. Genetic Differentiation and Signatures of Selection

To explore genetic divergence and potential selective pressures, we analyzed genetic differentiation (FST), selective sweeps (XP-CLR), and neutrality (Tajima’s D) between the bud sport and conventional populations (Figure 3D–F).

Pairwise FST analysis revealed multiple genomic regions with significantly elevated differentiation (Figure 3D), highlighting loci that have substantially diverged between the two populations. These highly differentiated regions may harbor genes underlying the distinct horticultural traits of bud sports.

Genome-wide scanning with XP-CLR identified several regions with high scores in the bud sport population, indicating potential selective sweeps [32] (Figure 3E). These regions are candidates for positive selection, potentially associated with novel horticultural traits arising from somatic mutation.

Tajima’s D analysis showed negative mean values for both populations, consistent with population expansion or purifying selection [33]. However, the bud sport population exhibited a significantly less negative Tajima’s D than the conventional population (*p* < 0.0001; Figure 3F), suggesting that the conventional population experienced a stronger or more recent expansion, or maintains more low-frequency variants. The less negative value in bud sports aligns with their origin from a limited number of somatic mutants and subsequent clonal propagation.

### 3.6. KEGG Enrichment and Differential Mutated Gene Analysis

**Figure 4:**
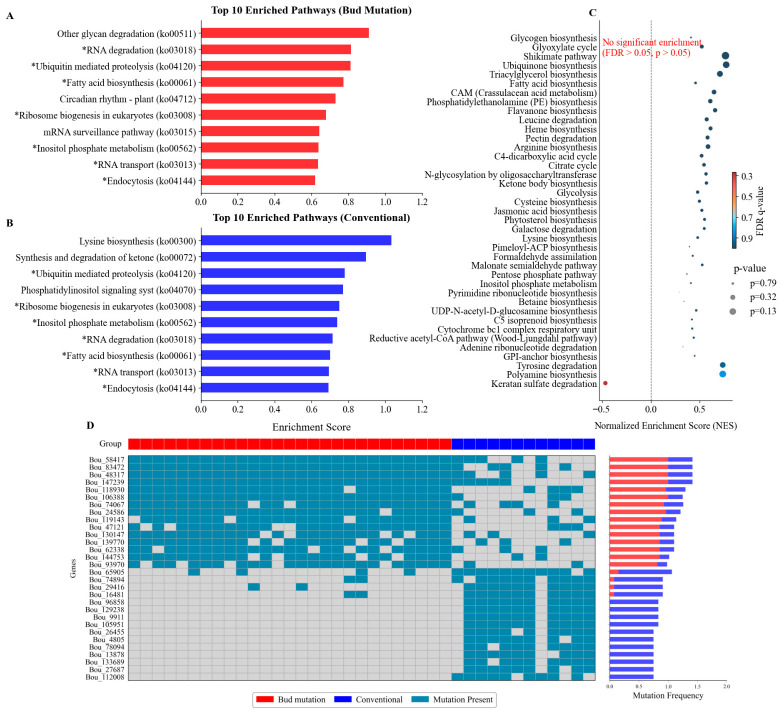
Comparative KEGG Enrichment and mutational landscape between bud sport and conventional populations. (**A**,**B**) KEGG pathway enrichment analysis of the top 10 significantly enriched pathways in the (**A**) bud sport and (**B**) conventional populations, respectively; pathways labeled with an asterisk (*) are common to both populations. (**C**) Gene Set Enrichment Analysis (GSEA) compares pathway enrichment patterns between the two populations. The *x*-axis represents the Normalized Enrichment Score (NES), and the *y*-axis shows the signaling pathways. The size of each point corresponds to the statistical significance (−log10(*p*-value)), and the color indicates the false discovery rate (FDR). (**D**) Distribution of genes with significant allele frequency differences between bud sport and conventional populations. The central heatmap (cyan) denotes the mutation status (presence/absence) of each gene in the respective groups. The adjacent bar plot illustrates the mutation frequency of each gene, with red and blue bars representing the bud sport and conventional groups, respectively.

To elucidate the biological processes underlying the differentiation between the bud sport and conventional populations, functional annotation of the detected genetic variations was performed, followed by KEGG pathway enrichment analysis of the top 10 most enriched pathways in each population. The results revealed 7 shared enriched pathways between the two groups, accounting for 70% of the top 10 pathways in both the bud sport and conventional populations. These core pathways include ubiquitin-mediated proteolysis (ko04120), RNA degradation (ko03018), fatty acid biosynthesis (ko00061), as well as Ribosome biogenesis in eukaryotes (ko03008), Inositol phosphate metabolism (ko00562), RNA transport (ko03013), and Endocytosis (ko04144) (Figure 4A,B). However, no significant difference in enrichment levels was found between the populations for these pathways (FDR > 0.05; Figure 4C).

We identified individual genes with significant differences in mutation frequency between the groups (FDR < 0.01; Figure 4D, Table S3). Several genes showed markedly higher mutation frequencies in the bud sport population, including *Bou_106388* (DNA-binding protein), *Bou_24586* (60S ribosomal protein), *Bou_144753* (zinc finger protein), *Bou_93970* (SelT-like protein), and *Bou_119143* (UDP-rhamnose rhamnosyltransferase 1), with frequency differences exceeding 0.64. Among these, UDP-rhamnose rhamnosyltransferase (*Bou_119143*) is particularly noteworthy, as this enzyme mediates the rhamnosylation of betalain precursors or betalains [12,34], thereby regulating pigment stability and hue and potentially accounting for the altered bract color intensity observed in many bud sports.

Conversely, several genes had significantly higher mutation frequencies in the conventional population. Notably, the mutation frequencies of *Bou_96858*, *Bou_129238*, *Bou_105951* (E3 ubiquitin–protein ligase RFWD3), and *Bou_9911* were zero in bud sports in contrast to a high frequency of 0.83 in conventional varieties. Other genes exhibiting significantly higher frequencies in conventional populations included *Bou_65905* (transmembrane protein 64-like), *Bou_29416* (carboxylesterase), *Bou_27687* (cytochrome c oxidase subunit 5b), *Bou_133689* (actin-related protein 2/3 complex subunit 3), and *Bou_112008* (COP9 signalosome complex subunit 7). The absence of mutations in E3 ubiquitin ligase RFWD3 (*Bou_105951*) across all bud sport individuals suggests that maintaining normal function of this gene may be a recurring requirement during somatic mutation events.

The differentially mutated genes, spanning protein degradation, transcriptional regulation, metabolism, and cellular structure, suggest multiple potential mechanisms contributing to the phenotypic divergence between bud sport and conventional populations. These results indicate that while major developmental pathways are conserved, mutations in a limited set of regulatory and metabolic genes (e.g., UDP-rhamnosyltransferase, ubiquitin ligases) may drive the distinct ornamental traits of bud sports.

## 4. Discussion

The genomic era has provided unprecedented tools for deciphering the origin and evolution of ornamental plants. Our super-GBS analysis of a *Bougainvillea* population comprising both a core bud sport group derived from *B. × buttiana* ‘Miss Manila’ and a control group of conventionally bred varieties offers a comprehensive perspective on the genetic underpinnings of bud sports, highlighting their critical and irreplaceable role in the breeding of this species.

### 4.1. Unique Genetic Lineage Shaped by Asexual Reproduction and Population Bottleneck

Multi-dimensional population genetic analyses, including principal component analysis (PCA), ADMIXTURE-based population structure inference, and neighbor-joining (NJ) phylogenetic tree construction, consistently revealed that the *B. × buttiana* ‘Miss Manila’ bud mutation population constitutes a genetically distinct and cohesive lineage, exhibiting significant genetic differentiation from conventional non-bud mutation cultivars (Figure 2). This pattern is highly consistent with the known horticultural breeding history of bud sports: all these bud sports originated from somatic mutations in the single parental cultivar *B. × buttiana* ‘Miss Manila’ and were subsequently propagated asexually via cuttings or grafting. Analogous patterns featuring decreased genetic diversity and well-defined genetic clustering have been documented in clonal organisms, including the asexual plant Taraxacum officinale [35] and the unisexual fish Poecilia formosa [36], where somatic mutations yielded new genetic lineages with limited genetic divergence. Strict asexual reproduction inherently restricts gene flow and the accumulation of novel genetic variations [37], resulting in a significantly narrower genetic base of the bud mutation population compared to conventional cultivars (Figure 1E).

Quantitatively, the bud mutation population exhibited significantly lower nucleotide diversity (π), expected heterozygosity (He), and polymorphism information content (PIC) (*p* < 0.001), typical genomic signatures of a population that experienced a bottleneck followed by asexual expansion [38]. This pattern contrasts sharply with sexually reproducing endangered species such as Equus ferus przewalskii and golden snub-nosed monkeys, in which balancing selection on key functional loci (e.g., immune and reproductive genes) maintains elevated polymorphism at specific genomic regions, rather than the genome-wide reduction in diversity seen in our asexual bud mutation population [39,40]. The substantial difference in private alleles further corroborates this conclusion: the conventional population contained 23,797 private alleles, whereas the bud mutation population had only 335 (*p* < 0.0001; Figure 3B). This sharp reduction in private alleles is consistent with recent genomic studies documenting depleted private alleles in asexual and clonal lineages, including the Japanese knotweed complex [41] and the oribatid mite *Oppiella nova* [42], a conserved genomic signature of clonal populations. However, it is worth noting that the magnitude of private allele loss in our bud sport population (over 98% reduction) is more extreme than that reported in some other clonal systems, possibly due to the particularly narrow founder base of ‘Miss Manila’. Despite the sterility of most *Bougainvillea* cultivars, conventional populations likely originated from multiple genetic backgrounds and occasionally undergo outcrossing, enabling them to maintain a relatively diverse gene pool and facilitate the accumulation of unique alleles [3,11]. In contrast, as asexual lineages, bud sports inherit only a limited set of alleles from their parental cultivar, with novel variations arising solely from rare somatic mutations. This explains the scarcity of private alleles and reduced heterozygosity in the bud mutation population (Figure 3C).

Notably, ADMIXTURE analysis revealed that the ancestry components of two conventional germplasms (SJM145-1 and SJM013-1) exhibit overlap with those of the bud mutation clusters (POP2 and POP4) (Figure 2C). This observation is reminiscent of findings in diverse plant groups, including grapevine and rice, where ADMIXTURE-based analyses have documented extensive ancestry overlap and admixture between wild and domesticated lineages [43,44]; in our case, it suggests a potential phylogenetic relationship between them, implying they may have originated from a common ancestor. However, an alternative explanation is that these conventional accessions have retained ancestral polymorphisms that were also present in the progenitor of ‘Miss Manila’, rather than indicating recent gene flow. The result indicates that the evolutionary history of *bougainvillea* germplasm resources remains complex, even within seemingly distinct clusters.

### 4.2. Evolutionary Dynamics: Selection Signals and Population History

Although asexual reproduction constrains the variation potential of bud mutation genomes, our analyses reveal that the evolution of these genomes is not a random process; rather, it is jointly shaped by population history and selection pressures. Pairwise FST analysis identified several genomic regions with significantly elevated genetic differentiation between bud mutation and conventional varieties (Figure 3D), suggesting that divergence at these loci may be driven by genetic drift or selection [45]. Complementing this, XP-CLR scanning uncovered distinct signatures of selective sweeps within the bud mutation population (Figure 3E), indicating that these genomic regions have potentially undergone positive selection [26]. Similar selective sweep signatures associated with domestication traits have been reported in other ornamentals, such as rose petal number [46] and chrysanthemum plant height [47]. This selection is hypothesized to be linked to artificial selection for favorable horticultural traits during domestication and cultivation, such as bract coloration, plant architecture, and flowering phenology. Nevertheless, we acknowledge that the current data do not directly prove selection; alternative forces such as background selection or demographic effects could also produce similar patterns [48]. Collectively, these findings are consistent with the notion that bud mutations are not entirely random in their evolutionary trajectory: directional artificial selection for novel and favorable phenotypes acts to screen and fix beneficial somatic mutations within asexual lineages, potentially leading to the formation of the selective sweeps observed in our genomic analyses.

Tajima’s D analysis further revealed distinct demographic histories between the two populations. Both groups showed negative mean Tajima’s D values (Figure 3F), consistent with population expansion or purifying selection [49,50]. However, the bud mutation population exhibited a significantly less negative Tajima’s D (*p* < 0.0001; Figure 3F). This pattern aligns with the evolutionary model for bud mutation populations: such mutations arise from a small set of somatic mutants that subsequently undergo clonal propagation. In this scenario, clonal expansion constrains the introduction of new low-frequency genetic variants, thereby preventing the excessive accumulation of rare alleles that would otherwise shift Tajima’s D to more negative values. A similar trend of less negative Tajima’s D in clonally propagated versus seed-propagated populations has been observed in cassava [51] and potato [52], supporting the generalizability of our findings. In contrast, the more negative Tajima’s D values observed in the conventional population imply either historical population expansion or a higher abundance of rare variants [53]. These patterns align with the population’s genetically diverse background and the potential for occasional outcrossing events.

Together, these results highlight the divergent evolutionary trajectories of the two groups: bud mutations represent a young, genetically constrained lineage governed by asexual reproduction and artificial selection, whereas conventional varieties constitute an ancient, dynamic population with a more complex demographic history.

### 4.3. Functional Implications of Differentially Mutated Genes

This study identified genes with significantly different mutation frequencies between bud sport and conventional varieties, offering preliminary insights into the molecular basis of their phenotypic differentiation. These genes span diverse functional domains, including pigment biosynthesis, transcriptional regulation, protein degradation, and cellular metabolism, indicating that the development of bud sport-specific traits involves the synergistic action of multiple pathways (Figure 4D).

A particularly notable finding is the significantly elevated mutation frequency of the *Bou_119143* gene (encoding UDP-rhamnose rhamnosyltransferase 1) in the bud sport population. As a member of the Caryophyllales, *Bougainvillea* produces betalains (rather than anthocyanins) as the primary pigments responsible for bract coloration [12]. While this enzyme is traditionally known to catalyze the glycosylation of flavonoids [54,55,56,57], emerging evidence indicates that UDP-rhamnose-dependent glycosyltransferases are highly likely to modify betalain precursors or betalain [12,34]. This glycosylation represents a key post-translational modification that regulates the stability, water-solubility, and intracellular localization of betalains, thereby directly modulating pigment intensity and color variation. Given that bract color is a primary target of selection in *Bougainvillea* bud sports, mutations in *Bou_119143* might alter the glycosylation pattern of betalains and flavonoids, potentially contributing to the diverse bract color phenotypes (e.g., pink, red, and purple) observed in the *B. × buttiana* ‘Miss Manila’ bud sport lineage. Nevertheless, direct biochemical evidence linking the specific mutations to altered enzyme activity is currently lacking and should be addressed in future studies. Similarly, the increased mutation frequencies of genes involved in transcriptional regulation (e.g., *Bou_106388*, encoding a DNA-binding protein, and *Bou_144753*, encoding a zinc finger protein) suggest that alterations in gene regulatory networks could drive broader phenotypic variation in important horticultural traits such as plant architecture, branching pattern, and flowering time [58,59,60].

Conversely, several genes were completely devoid of mutations in the bud sport population despite reaching mutation frequencies up to 83% in conventional varieties. These include *Bou_105951* (encoding the E3 ubiquitin–protein ligase RFWD3) and *Bou_112008* (encoding COP9 signalosome subunit 7). This pattern implies the existence of genetic constraints specific to the asexual *B. × buttiana* ‘Miss Manila’ lineage. E3 ubiquitin–protein ligases are central regulators of protein turnover, cell cycle progression, and stress responses [61]. The COP9 signalosome regulates the activity of cullin-RING ligase (CRL) families of E3 ubiquitin ligase complexes, and plays critical roles in regulating gene expression, cell proliferation, and cell cycle [62]. Consequently, mutations in these essential pathways may be incompatible with the survival or adaptability of this clonal background. Their elimination in bud sports underscores the possible action of purifying selection in pruning deleterious genetic variation from the asexual genome.

### 4.4. Pathway-Level Analysis and Phenotypic Implications

Comparative KEGG pathway enrichment analysis of the top 10 ranked pathways for each population revealed a landscape of shared core functions alongside group-specific signatures. Seven pathways were common to both groups, including “ubiquitin-mediated proteolysis,” “RNA degradation,” “Ribosome biogenesis in eukaryotes,” “fatty acid biosynthesis,” “Inositol phosphate metabolism,” “RNA transport,” and “Endocytosis.” This substantial overlap underscores a conserved foundation of essential cellular processes related to protein homeostasis, RNA metabolism, and basic cellular functions.

Despite this common core, each population exhibited distinct pathway enrichments. The bud sport population uniquely featured “other glycan degradation,” “mRNA surveillance pathway,” and “circadian rhythm-plant.” These specificities may be more directly linked to observed phenotypic divergence. For instance, enhanced “glycan degradation” could influence cell wall dynamics or pigment modification [63,64], while alterations in “circadian rhythm” pathways are known to affect key horticultural traits like flowering time and photomorphogenesis [65]. In contrast, the conventional population uniquely enriched pathways such as “lysine biosynthesis,” “synthesis and degradation of ketone bodies,” and “Phosphatidylinositol signaling system,” reflecting a broader engagement with primary metabolism and complex signal transduction [66,67,68], a pattern consistent with its more diverse genetic background and sexual reproductive history.

Crucially, while these overlapping core pathways were significantly enriched in both groups, their enrichment levels did not differ statistically between the populations (Figure 4C). This key result indicates that the major phenotypic differentiation between bud sports and conventional varieties is not driven by large-scale changes in the utilization of fundamental biological pathways. Instead, it is consistent with a model where targeted mutations within specific genes of these conserved pathways, such as *Bou_119143* in the modification of betalains and flavonoids, complemented by the influence of the population-specific pathways noted above. Therefore, we suggest that bud sport variation arises predominantly from precise genetic modifications in key functional nodes against a backdrop of conserved core cellular machinery, rather than from a wholesale reorganization of metabolic or regulatory networks.

### 4.5. Limitations and Future Research Directions

While this study provides insights, some limitations exist. Although a chromosome-level reference genome of *B. glabra* has been published [11], it lacks a complete gene annotation file, precluding its direct use for function-related analyses. Consequently, this study ultimately adopted the scaffold-level reference genome of *B. × buttiana* “Mrs. Butt” with available gene annotations for analysis [12]. However, the scaffold-level assembly characteristics (Figure 3A) still limited the fine mapping of selective sweep regions and the precise chromosomal anchoring of mutated genes, hindering further improvement in the resolution of relevant genetic analyses. Future genome assemblies with improved contiguity and annotation will be essential to overcome this limitation. Additionally, candidate genes require validation through functional assays such as expression analysis or genetic transformation. Future work integrating precise phenotypic data with genomic approaches like GWAS would help definitively link genetic variants to key horticultural traits. Given the limited sample size of the conventional group (n = 12), GWAS power may be low; expanding the collection would be beneficial. We also note that all statistical comparisons assumed independence of accessions, but clonal relatedness within the bud sport group could inflate type I error rates; permutation-based methods or mixed models accounting for kinship would be valuable in future analyses.

## 5. Conclusions

This study analyzed 39 *Bougainvillea* accessions using genotyping by sequencing (GBS). It revealed that the bud sports of ‘Miss Manila’ are clonal lineages with a narrow genetic basis shaped jointly by somatic mutations, artificial selection, and purification selection. These bud sports exhibit significant genetic differentiation from conventional cultivars and a distinct population structure. Meanwhile, we identified selected genomic regions and high-frequency mutated genes, which are consistent with the view that bud sport breeding is an efficient approach for *Bougainvillea* germplasm innovation. This research deepens our understanding of the mechanism underlying bud sport formation and establishes the core role of bud sports in *Bougainvillea* genetics.

## Data Availability

The datasets generated and analyzed during the current study are available in the National Genomics Data Center (NGDC) Genome Sequence Archive (GSA) repository, under accession number CRA035256 (https://ngdc.cncb.ac.cn/gsa/browse/CRA035256, accessed on 16 April 2026).

## Supplementary Materials

The following supporting information can be downloaded at: https://www.mdpi.com/article/10.3390/genes17040471/s1, Table S1: basic information of Bougainvillea used in the study; Table S2: the sequencing data for 39 *Bougainvillea* ‘Miss Manila’ samples; Table S3: significantly differentiated genes between bud sport and conventional populations.

## Abbreviations

The following abbreviations are used in this manuscript:

GBS	genotyping-by-sequencing
PCA	principal component analysis
He	expected heterozygosity
Ho	observed heterozygosity
PIC	polymorphism information content
Pi	nucleotide diversity
FDR	false discovery rate
GSEA	Gene Set Enrichment Analysis
